# Supplementary figures and images for: Diagnostic performance and usability of the VISITECT CD4 semi-quantitative test for advanced HIV disease screening
Source: PLoS One. 2020 Apr 3;15(4):e0230453. doi: 10.1371/journal.pone.0230453 (PMC7122771; doi:10.1371/journal.pone.0230453)

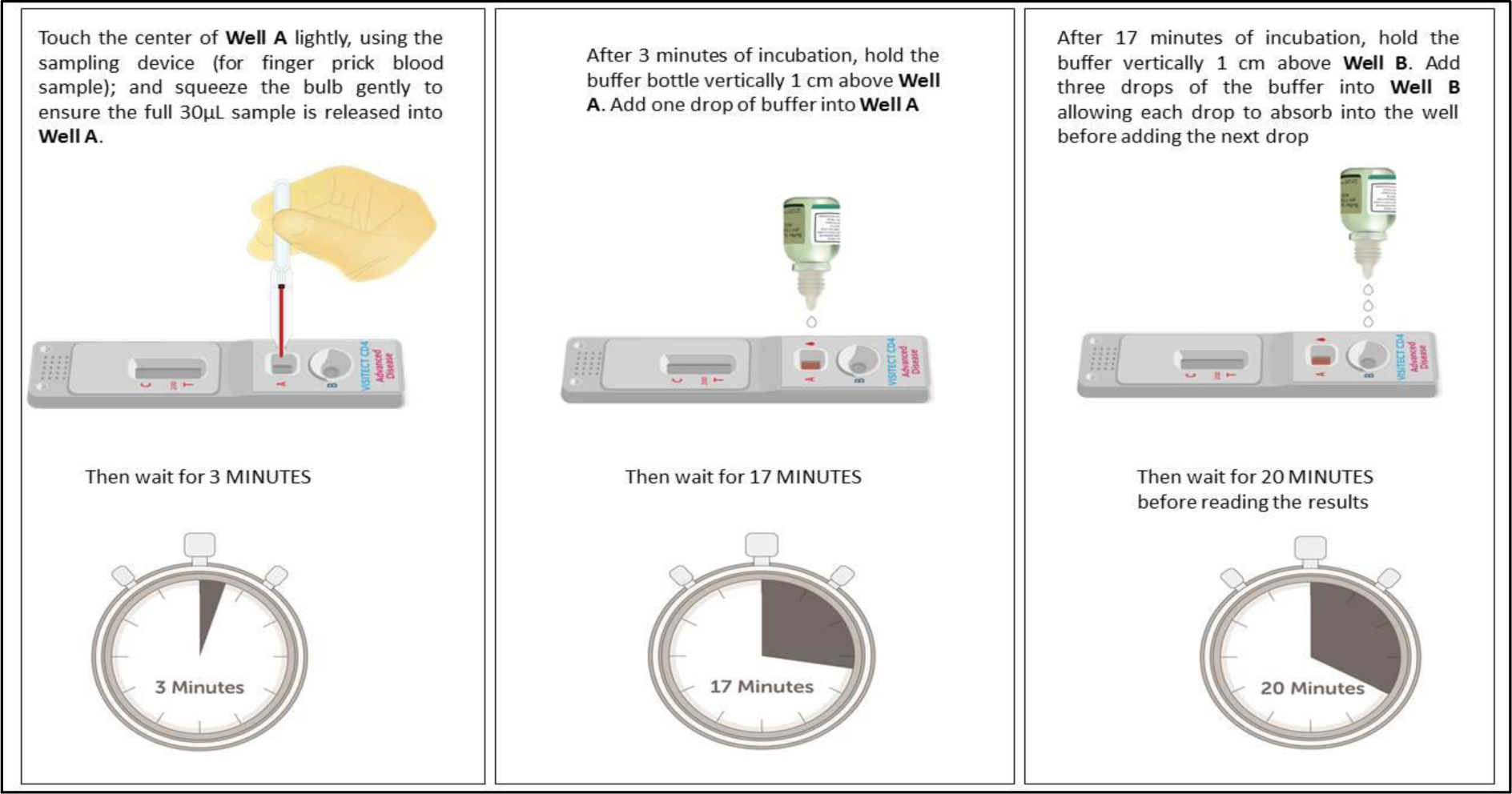

Supplement: S1 Appendix — (TIF) [file pone.0230453.s001.tif]

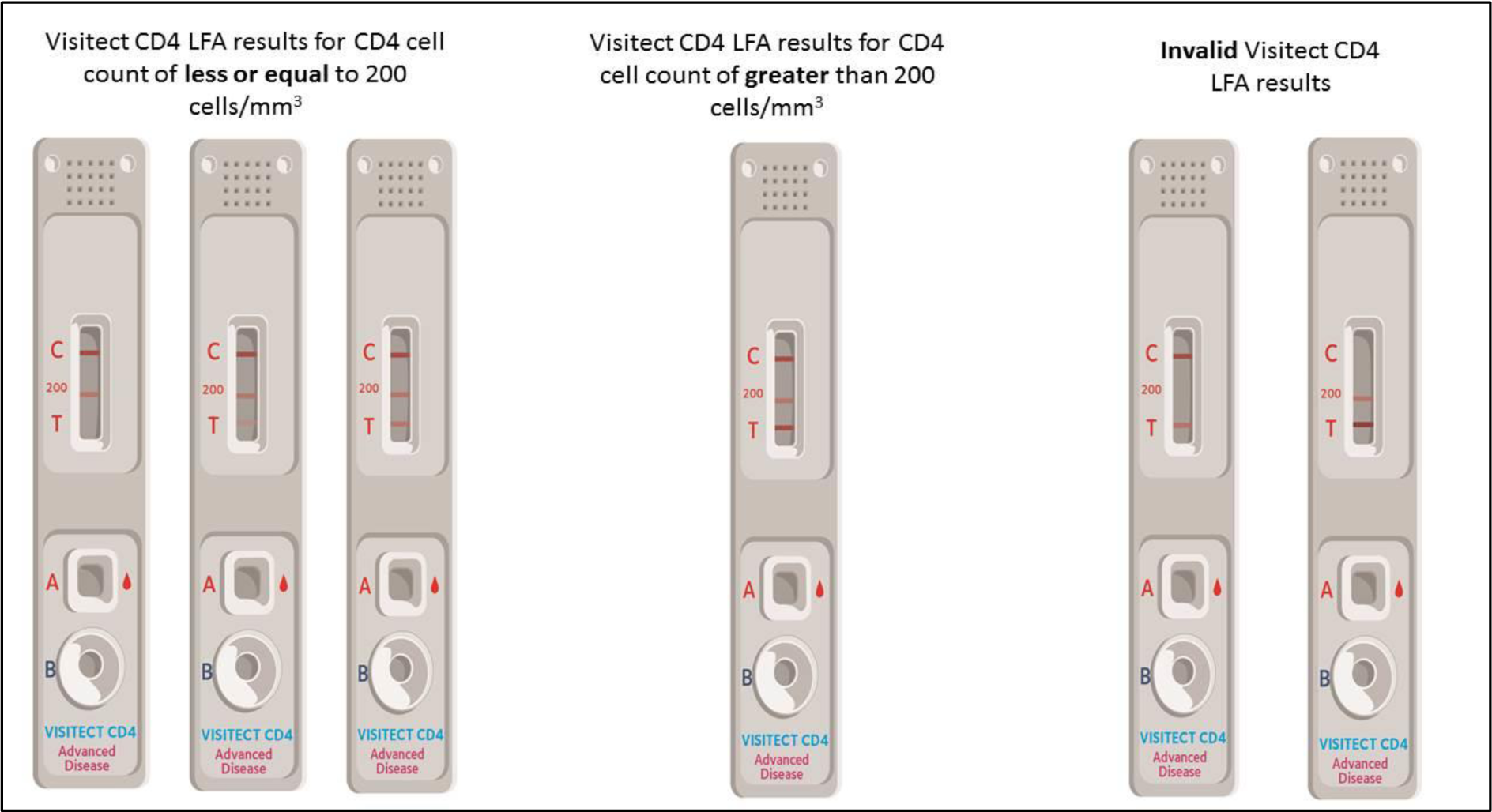

Supplement: S2 Appendix — (TIF) [file pone.0230453.s002.tif]
