# Supplementary material for: Diagnostic performance and usability of the VISITECT CD4 semi-quantitative test for advanced HIV disease screening
Source: PLoS One. 2020 Apr 3;15(4):e0230453. doi: 10.1371/journal.pone.0230453 (PMC7122771; doi:10.1371/journal.pone.0230453)
Supplement: Attachment — Submitted filename: Rebutal letter_PONE-D-19-30572.doc [file pone.0230453.s003.doc]

We would like to thank the reviewers for their time and concise inputs.

This study was initially submitted to PLOS-One with results from two study sites (Malawi and DRC); however, the study in Zimbabwe has also been finished recently and we have herein added the results from Zimbabwe for 118 patients tested in Phase 1. We also added the co-investigator details from Zimbabwe and also added the Ethical Approvals obtained from the Zimbabwe Medical Research Council.

**Responses to reviewer comments**

**Reviewer #1:**

Reviewer comment 1: I suggest including your reference method in the abstract.

**Author response**: line 66 and 67 of the abstract do mention the reference test method used in the study (i.e BD FACSCount assay for CD4 testing)

Reviewer comment 2: On line 176 of the methods section, the following sentence was cut-off “The Visitect CD4 LFA test kit can be stored”

**Author response**: We have corrected the sentence and it now reads; “The Visitect CD4 LFA test kit can be stored within temperature ranges of 2 - 30^o^C” (line 195).

Reviewer comment 3: When assessing the performance of Visitect CD4 LFA at different reference test cut-offs, I recommend including the number of samples compared at each cut-off.

**Author response**: We have added the number and percentage of samples for each of the cut-off ranges (line 248, table 3)

Reviewer comment 4: On line 223, the authors state “In restricting the analysis to samples with CD4 < 100 cellsmm3 cut-off in the reference test, the sensitivity of the Visitect CD4 LFA was 98% [IQR: 93.1 – 99.8] and specificity was 66.6% [IQR: 62.2 – 70.8].” If the analysis was truly restricted to reference values < 100 cells/mm3 then there would be no false-positives from the test assay because there would be no reference values > 200 cells/ml. Was this analysis instead performed by changing the cut-off of the reference assay? I recommend a better explanation of this analysis here or in the methods section.

**Author response**:

We have modified the sentence to reflect that the sensitivity/specificity of Visitect CD4 LFA at the different cut-offs is purely based on the reference assay cut-offs, whilst the Visitect CD4 LFA remains with its standard threshold of 200cells/mm^3^. Line 242-244. Furthermore, we avoid mentioning the words ‘false positive/false negative’ during the interpretation of the results of this restriction analysis.

Reviewer comment 5: The authors shared the Kappa statistic of the Visitect CD4 LFA compared to the Pima CD4, but not the specificity or sensitivity. I recommend sharing the sensitivity and specificity with the Pima CD4 as the reference assay, or an explanation of why the authors chose to use venous blood tested on the BD FACScount as the reference assay for FP samples tested on the Visitect CD4 LFA.

**Author response**: we used the Kappa statistic to compare diagnostic performance of Visitect CD4 LFA to PIMA (and not the sensitivity/specificity) because PIMA is not a reference standard method for CD4 cell count testing, as such Kappa statistic is the most correct/recommended way to assess the agreement between two methods (where there is no reference/gold standard) as both are considered imperfect tests.

**Reviewer #2**:

Reviewer comment 1: The authors use sensitivity and specificity to describe the correlation between the different methods at the threshold of 200, which introduces some confusion as compared with test evaluations that compare positives and negatives as is done in most method comparisons. It might be more clear to describe the relationship as a correlation or concordance of methods at >200 or <200. Alternatively, it could be stated as the sensitivity or accuracy to detect <200 and the sensitivity or accuracy to detect >200.

**Author response**: Yes, we understand the potential ‘*confusion’* that could arise with interpreting results for such a binary-test which doesn’t necessarily provide the traditional ‘positive’ or ‘negative’ results. The consequence is that Visitect CD4 LFA results that are <200 can loosely be called ‘positive’ whilst those >200 can be said to be ‘negative’.

In our study and in data analysis packages, as long as diagnostic result outcomes are dichotomous/binary, they are automatically labeled as ‘normal’ and ‘abnormal’, which enables determination of sensitivity/specificity to a reference standard method. This is regardless of whether the result is positive/negative or is <200/>200. Nonetheless, in the manuscript, we do emphasize (in multiple occasions) that the calculated outcomes of performance are sensitivity/specificity to detect CD4 cell counts at the 200cells/mm^3^ threshold (meaning either >200 or less than 200), line 230-232.

Reviewer comment 2: Precision studies: Were any samples used that produced an equal result intensity with the 200 line? That is, precision studies should include a sample near the cutoff where changes could be realized. The nature of the samples used is not described in the Methods Section.

**Author response**: Line 199-201 in Methodology section does partly mention the strategy for assessing the precision of the index test. In line 250-253 (results section), we do mention the outcomes of precision assessment, and the fact that we used 12 samples and 2 had CD4 cell counts close to the 200cells/mm^3^ threshold. Nonetheless, we certainly do acknowledge the need to describe the samples used for precision; however, since we used a range of samples (in terms of CD4 cell counts) we could not manage to describe all the sample types used within the methodology/results section.

Reviewer comment 3: Reproducibility study: What is described is not a reproducibility study; it describes an experiment where incubation times are varied and the effect on results is observed. The authors should consider to remove this because it is small in scope, not relevant for the user because they are supposed to follow instructions, and it might give the impression that incubation times can be varied without having an effect on test results. If it were to be included, then perhaps there should be other investigations also (e.g., varying the number of drops of buffer, using partial volumes of sample, etc.) to be more complete. A true reproducibility study shows how the test performs with multiple testing of the same samples over hours, days, or longer, and with varying conditions (different users, different temperatures on days, etc.).

**Author response**: We acknowledge that our reproducibility part of the study is limited in scope and the idea behind it was purely to explore the effect of varying the incubation times only; as we pragmatically know that this will be one of the biggest independent factors to affect test results in different real user settings. Practically, we know health-care workers sometimes do not follow the instructions for use (IFU) for many POC diagnostics and with this multi-step test; we sought to show the potential consequences of not following the instructions. We do acknowledge that our findings may be taken to mean that the incubation times can be changed, but we specifically sought to provide a clue on this question that these incubation times are un-forgiving and testers must adhere to manufacture times. To simplify the text in the manuscript, we have removed part of the reproducibility data (Table 4) to ensure limited information remains; and the Discussion section (line 339-347) provide a clear emphasis on following manufacture incubation times.

Reviewer comment 4: Line 176 is a partial sentence.

**Author response**: We have corrected the sentence and it now reads; “The Visitect CD4 LFA test kit can be stored within temperature ranges of 2 - 30^o^C” (line 195).

Reviewer comment 5: Line 253 - the ambient temperature should be stated (in some resource limited countries it could be 35C). Stability could also mean how the test performs after storage at different temperatures over time. Therefore, it should refer to "stability of the observed results".

**Author response**: we have modified this and clarified that the ambient laboratory temperature range was 24–28^o^C. Line 269 Furthermore, we have added the word ‘result re-reading’ in the subtitle (line 268) to emphasize that the stability results are just from re-reading the stored test cassettes only.

Reviewer comment 6: In the early years of 2000, there was a method called TRAxCD4 (T Cell Dxs/Innogenetics) that was an ELISA based method that measured soluble CD4 protein in a sandwich configuration (similar to the principle in this LFA). The authors should review the literature and include any references in their manuscript.

**Author response**: We have done a literature review of this TRAXCD4 technology and many others during the early 2000 and we have added a reference of one of the review articles on this (reference number 7). We however, did not add the whole technology appraisal from early 2000s in detail in the manuscript as most of these technologies are obsolete. Also we wanted our Introduction Section to focus in the currently commercially available point-of-care (POC) or near-POC CD4 assays in the market (line 110-113).
